# Co-Infection with *Anaplasma* Species and Novel Genetic Variants Detected in Cattle and Goats in the Republic of Korea

**DOI:** 10.3390/pathogens10010028

**Published:** 2021-01-01

**Authors:** Evelyn Alejandra Miranda, Sun-Woo Han, Yoon-Kyong Cho, Kyoung-Seong Choi, Joon-Seok Chae

**Affiliations:** 1 Laboratory of Veterinary Internal Medicine, BK21 PLUS Program for Creative Veterinary Science Research, Research Institute for Veterinary Science and College of Veterinary Medicine, Seoul National University, 1 Gwanak-ro, Gwanak-gu, Seoul 08826, Korea; evelyn_ale08@hotmail.com (E.A.M.); suntina227@snu.ac.kr (S.-W.H.); hmhm0410@snu.ac.kr (Y.-K.C.); 2 College of Ecology and Environmental Science, Kyungpook National University, Sangju 37224, Korea; kschoi3@knu.ac.kr

**Keywords:** *Anaplasma phagocytophilum*, *Anaplasma bovis*, *Anaplasma capra*, cattle, goat, co-infection, Republic of Korea

## Abstract

Anaplasmosis, a tick-borne disease with multiple reservoirs, has been evolving in its pathogenesis, increasing domestic ruminants susceptibility to simultaneous infections with multiple pathogens. However, there is limited information regarding anaplasmosis in domestic ruminants in the Republic of Korea (ROK). We aimed to evaluate the role of Korean cattle and goats in *Anaplasma* infection maintenance. Polymerase chain reaction was performed to investigate the prevalence and genetic diversity of *Anaplasma* spp. from 686 whole blood samples collected from different ROK provinces. *Anaplasma* infection was mostly caused by *A. phagocytophilum* (21.1%) in cattle, and *A. bovis* (7.3%) in goats. Co-infection cases were found in cattle: *A. bovis* and *A. phagocytophilum* (16.7%), and in goats: *A. bovis* and *A. capra* (1.0%). Notably, a triple co-infection with *A. bovis*, *A. phagocytophilum*, and *A. capra* was found in one cow. Phylogenetic analysis revealed novel variants of the *A. phagocytophilum 16S rRNA* and *A. capra* *gltA* genes. This research contributes to the ratification of cattle as a potential reservoir of *A. capra* and demonstrates *Anaplasma* co-infection types in Korean domestic ruminants. As anaplasmosis is a zoonotic disease, our study could be crucial in making important decisions for public health.

## 1. Introduction

Anaplasmosis, an infectious but non-contagious tick-borne disease, classically related as a disease of ruminants, is caused by obligate intra-erythrocytic bacteria of the genus *Anaplasma* [1] discovered in 1910 by Theiler [2]. This genus belongs to the *Anaplasmataceae* family and is composed of six confirmed species based on host cell tropism: *A. phagocytophilum*, *A. bovis*, and *A. platys*, which infect neutrophils [3], monocytes [4], and platelets [5], respectively, whereas *A. marginale*, *A. centrale*, and *A. ovis* infect erythrocytes [6]. Furthermore, the newly recognized *Anaplasma* species, *A. capra* might infect endothelial cells [7].

Anaplasmosis in domestic ruminants is caused by two main etiological agents: *A. marginale* in cattle [8] and *A. ovis* in sheep and goats [9]. However, additional studies on the genus *Anaplasma* have detected other *Anaplasma* spp. [10] that may act as a causative agent of anaplasmosis in ruminants. In the Republic of Korea (ROK), *A. phagocytophilum* [11] and *A. bovis* [12] infections have been recently detected in Holstein cattle. Moreover, a recent study that was conducted in this country suggests that cattle may serve as potential reservoirs of *A. capra* [13]. Currently, *A. centrale* has not been detected in the ROK [13], but this does not rule it out as a causative agent of bovine anaplasmosis, since a clinical case associated with this species has been reported in the European continent [14]. Regarding caprine anaplasmosis, in addition to *A. ovis* infection, *A. phagocytophilum* and *A. bovis* infections have been detected in goats from Central and Southern China [15] and *A. capra* infection in Korean native goats (*Capra aegagrus hircus*) [16].

Additionally, co-infection cases with *Anaplasma* species have been identified in Chinese domestic ruminants using polymerase chain reaction (PCR), such as *A. ovis* plus *A. bovis*, *A. ovis* plus *A. phagocytophilum*, and *A. bovis* plus *A. phagocytophilum* in goats [15], *A. bovis* plus *A. phagocytophilum* in cattle, and *A. ovis* plus *A. phagocytophilum* in sheep [17]. In the ROK, co-infection with *A. phagocytophilum* and *A. phagocytophilum*-like *Anaplasma* spp. has been detected in cattle [18].

Tick-borne zoonoses have been observed since the second half of the 19th century [19]. To date, there are three *Anaplasma* species that have been recognized as zoonotic pathogens: *A. phagocytophilum* [20], *A. ovis* [21], and *A. capra*, recently isolated from 28 human patients during acute phase illness in Heilongjiang, China [7]. In the ROK, serological evidence of *A. phagocytophilum* infection in human subjects with acute fever was first described in 2002, with a seropositivity rate of 1.8%. These results were also confirmed by western blotting and TaqMan real-time PCR [22]. Moreover, a recent study in the ROK reported a clinical case of human granulocytic anaplasmosis in a patient with a history of tick bite, which was confirmed by seroconversion, PCR, and sequence analysis of *A. phagocytophilum* [20].

*Anaplasma* spp. are mainly transmitted to humans and animals by specific species of ticks belonging to the *Ixodidae* family [23]. In the ROK, *A. phagocytophilum* and *A. bovis* infections have been identified not only in ticks of the *Haemaphysalis* genus, such as: *H. longicornis*, and *H. flava*, but also in ticks belonging to the *Ixodes* genus, such as: *I. persulcatus*, and *I. nipponensis* [24]. *H. longicornis* has been recognized as the most common tick species that infests Korean native goats [25] and Korean grazing cattle [26]. This arthropod vector is considered the most prevalent tick species associated with tick-borne pathogens, followed by *Ixodes* spp., throughout Korea [27].

Considering that ticks transmit more pathogenic species than any other group of blood-feeding arthropods worldwide [28], and given the increase in the human population along with the emergence of new animal species acting as reservoir hosts, it is expected that over the years, humans will become one of the most common blood sources for ticks [29], thus representing an impact on public health and a disadvantage to livestock farming due to the economic losses. The recognition of *Anaplasma* as a genus of public health significance has promoted an interest in these bacteria, translating into remarkable information about their molecular biology, genetics, and pathobiology [23]. However, in the ROK there is limited information regarding *Anaplasma* infection in domestic ruminants. Therefore, we aimed to investigate the prevalence and genetic variability of *Anaplasma* species circulating in cattle and goats according to geographic distribution to provide epidemiological information that could be crucial in making important decisions for animal and public health. 

## 2. Results

### 2.1. Distribution of Anaplasma Infection

**Figure 1:**
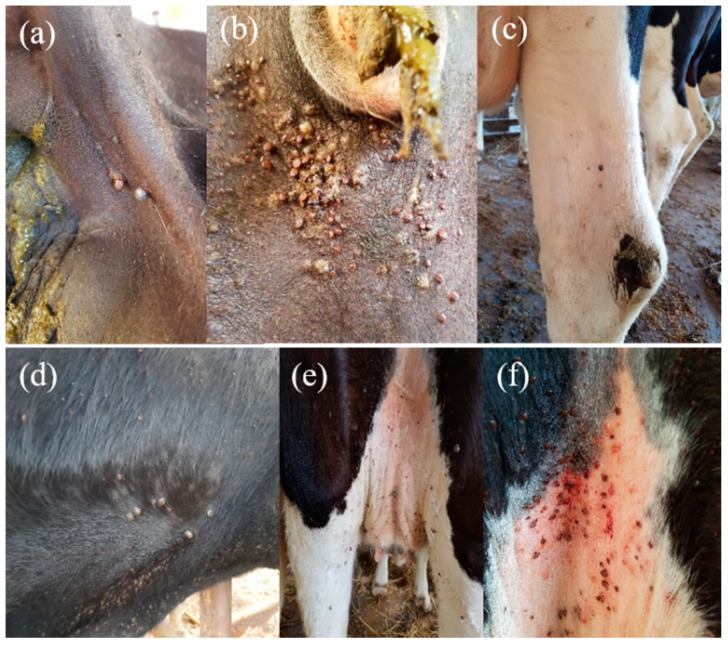
Cattle from the Gyeongsangbuk-do province with the highest *Anaplasma* spp. infection rates. A severe tick infestation was evident. (**a**) Ticks were attached behind the tail, (**b**) around the anus, where there was an accumulated number of ticks feeding on the host. (**c**) Ticks were also attached to the legs as well to (**d**) the dewlap; lesions caused by ticks were perceptible in this region. (**e**) Teats and (**f**) rear udder were the areas that presented the heaviest infestation, where different tick stages were evident. Additionally, local redness (red spots) and rashes were noticeable in those sites.

**Figure 2:**
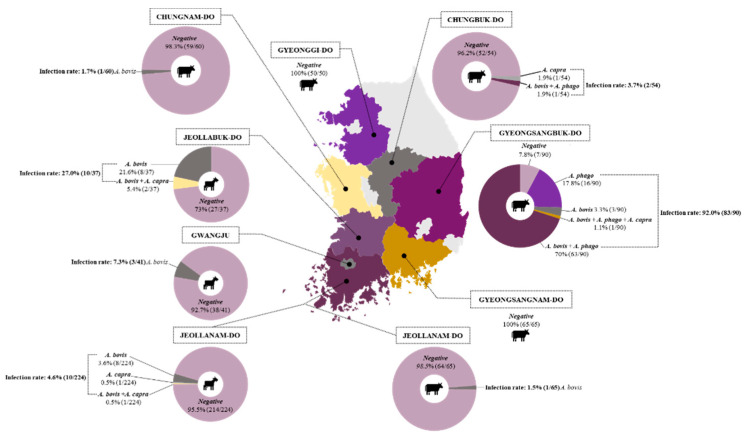
Geographical distribution of *Anaplasma* species in the provinces sampled in the Republic of Korea. Pie charts represent *Anaplasma* spp. infection rates for single infection and co-infection cases identified in each tested province. The cattle and goat icons indicate the sampled sites for respective animal species. *A. phago*: *Anaplasma phagocytophilum*.

*Anaplasma* infection in domestic ruminants was detected in five out of the seven tested provinces and one metropolitan city. The highest infection rate by causative agents of bovine anaplasmosis was found in the Gyeongsangbuk-do province (92.0%), where 83 out of 90 cattle were carriers of one or more *Anaplasma* species. In particular, the cattle tested in this province presented severe tick infestation, which were visible on different body parts of the animal (Figure 1). Chungbuk-do was reported to have an infection rate of 3.7%, followed by Chungnam-do and Jeollanam-do, which were provinces with a lower prevalence of *Anaplasma* species at 1.7% and 1.5%, respectively. In the Gyeongsangnam-do and Gyeonggi-do provinces, neither single infection nor co-infection cases were detected. In goats, the highest prevalence was observed in Jeollabuk-do (27.0%), followed by Gwangju Metropolitan City (7.3%) and Jeollanam-do province (4.6%), which had the lowest infection rates (Figure 2). 

### 2.2. Anaplasma Species Prevalence: Single Infection and Co-Infection Cases

As described in Table 1, 16 out of 384 cattle blood samples tested positive for *A. phagocytophilum* (4.2%), which was the dominant single infection among the analyzed *Anaplasma* species, followed by *A. bovis* with 1.3% (5/384). Single infection with *A. capra* occurred in only one cow (0.3%) from the Chungbuk-do province. *A. ovis* infection was not found in any of the tested cattle blood samples. Interestingly, co-infection with two pathogens (*A. bovis* and *A. phagocytophilum*) was found in 64 cattle (16.7%). Additionally, a special case of triple infection caused by the pathogens *A. bovis*, *A. phagocytophilum*, and *A. capra*, was identified in only one cow (0.3%) from Gyeongsangbuk-do province. 

On the other hand, goats were infected with *A. bovis* and *A. capra*. The dominant species in all the single infection cases was *A. bovis* with 6.3% (19/302), followed by *A. capra*, which was present in 0.3% of the total number of goats analyzed. Neither *A. phagocytophilum* nor *A. ovis* was detected in the analyzed goat blood samples. In contrast with the cattle results, co-infection cases in goats were caused by *A. bovis* and *A. capra*, reaching an infection rate of 1.0% (3/302). These findings indicate that the overall infection rate per host was 22.7% (87/384) in cattle and 8.0% (23/302) in goats, as shown in Table 1. 

### 2.3. Total Number of Animals Infected per Anaplasma Species Analyzed

Infection rates of *Anaplasma* spp. were also calculated for each etiological agent. In the present study, 81 (21.1%) cattle were positive for *A. phagocytophilum*, 70 (18.2%) for *A. bovis*, and 2 (0.5%) for *A. capra*. On the other hand, 22 (7.3%) goats were positive for *A. bovis* and four (1.3%) for *A. capra* (Table 2). 

### 2.4. PCR and Molecular Identification

PCR amplification of the *16S rRNA* gene was successfully achieved for the identification of *A. phagocytophilum*, *A. bovis*, and *A. capra*, generating fragments of 925 bp, 547 bp, and 1499 bp, respectively. For multilocus genotyping of *A. phagocytophilum*, no samples were positive for ankyrin-related protein (*ank*A) and major surface protein 2 (*msp2*) gene fragments. Amplification of the *A. bovis* heat shock protein (*groEL*) gene was successfully achieved with a fragment length of 845 bp. Regarding *A. capra*, two cattle and four goats were positive for its *16S rRNA* gene (1499 bp) as well as for the citrate synthase (*gltA*) gene (594 bp). However, only one goat was positive for *groEL* gene (874 bp) and two goats were positive for the major surface protein 4 (*msp4*) gene fragment with a length of 656 bp.

### 2.5. Sequences and Phylogenetic Analysis

**Figure 3:**
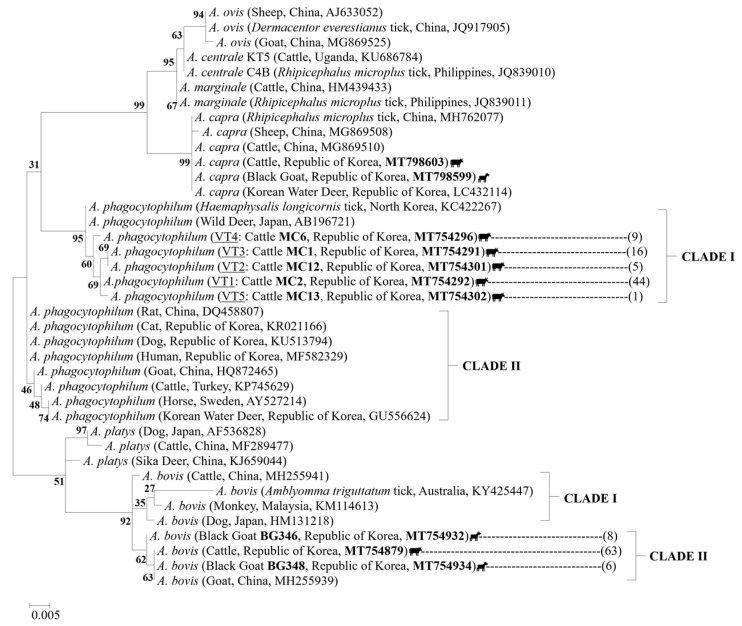
Phylogenetic tree based on the *16S rRNA* gene fragments of the *Anaplasma* species (547 bp). The sequence alignments were performed among the *A. phagocytophilum*, *A. bovis* and *A. capra* sequences obtained in this study and other members of the family *Anaplasmataceae*. The phylogenetic tree was constructed using the neighbor-joining method with 1000 replicates of the alignment (MEGA 6.06 software). Cattle and goat icons indicate the sequences found in this study for the respective animal species. Isolate, country, and GenBank accession numbers are shown in parentheses. Novel variants of *A. phagocytophilum* are indicated by the abbreviation VT (variant type). The numbers in brackets represent the total number of sequences that are identical to the representative sequence. The scale bar represents the number of nucleotide substitutions between sequences. Clades are denoted by roman numbers. Numbers on the branches indicate percent support for each clade.

To investigate the genetic variability of *A. phagocytophilum*, the 75 gene sequences obtained from cattle were analyzed and compared with sequences downloaded from the National Center for Biotechnology Information (NCBI). Analysis based on the *A. phagocytophilum 16S rRNA* gene fragments (925 bp) revealed the presence of five distinct strains, named variant type (VT): VT1 (*n* = 44), VT2 (*n* = 5), VT3 (*n* = 16), VT4 (*n* = 9) and VT5 (*n* = 1). The phylogenetic analysis demonstrated that these strains were classified into the first clade together with sequences previously isolated from North Korean *H. longicornis* ticks (KC422267) and Japanese wild deer (AB196721), sharing an identity percentage range of 98.0–99.6% and 96.3–99.6%, respectively (Figure 3).

**Figure 4:**
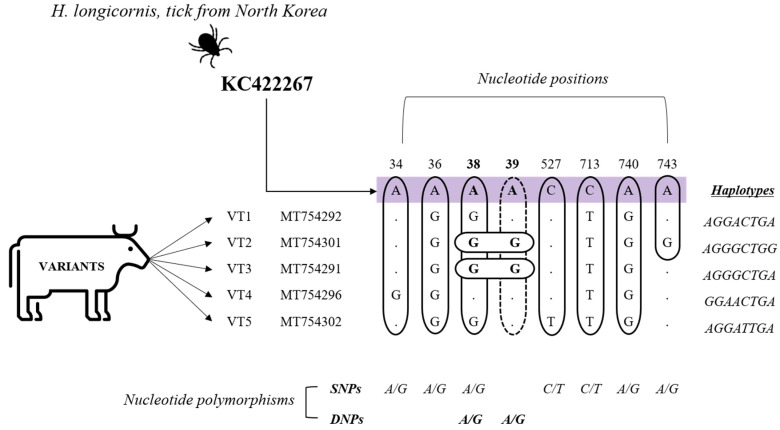
Novel variants of *Anaplasma phagocytophilum 16S rRNA* gene found in cattle. KC422267: reference sequence. Through the comparison between the reference sequence and the five variants identified in this study, single nucleotide polymorphisms (SNPs) were identified at positions 34, 36, 38, 740, and 743 with variation A/G; and positions 527 and 713 with variation C/T. Double nucleotide polymorphisms (DNPs) were found at positions 38 and 39 variation A/G, which are indicated by bold letters. The haplotypes generated due to the DNA variations found along the sequences are shown.

Further analyses of *A. phagocytophilum 16S rRNA* gene sequences revealed that the strains obtained in this study shared a high degree of identity (99.5–99.6%) with North Korean ticks isolates (Table S1); however, they presented single and double nucleotide polymorphisms, in which one or two consecutive nucleotides were altered compared with the reference sequence (KC422267; Figure 4), suggesting that all of them are novel variants of the *A. phagocytophilum 16S rRNA* gene.

The phylogenetic analysis of *A. bovis 16S rRNA* gene enabled classification of this *Anaplasma* species into two different clades. Clade I included isolates from China, Australia, Malaysia, and Japan, while clade II contained the 63 cattle sequences and the two different strains found in goat blood samples tested in this study [BG346 (*n* = 8) and BG348 (*n* = 6)], along with those from Chinese goats (MH255939; Figure 3), which were highly related, sharing an identity percentage of 100% with cattle isolates, 99.8% with variant BG346, and 100% with the variant BG348 (Table S2). The variant BG346 exhibited the highest sequence identity (100%) with *H. longicornis* tick isolates (GU064901) from the ROK.

**Figure 5:**
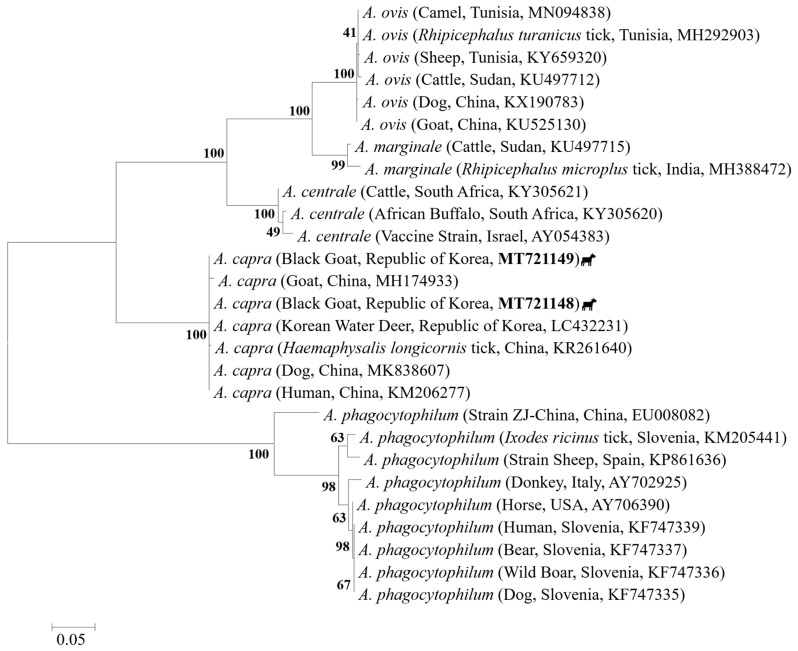
Phylogenetic tree based on the *msp4* gene fragments of the *Anaplasma* species (527 bp). The sequence alignments were performed among the *A. capra* sequences obtained in this study and other members of the family *Anaplasmataceae*. The phylogenetic tree was constructed using the neighbor-joining method with 1000 replicates of the alignment (MEGA 6.06 software). Goat icons indicate the sequences found in this study. Isolate, country, and GenBank accession numbers are shown in parentheses. The scale bar represents the number of nucleotide substitutions between sequences. Numbers on the branches indicate percent support for each clade.

*A. capra 16S rRNA* sequences obtained from cattle and goats showed 100% identity with the sequences detected in *Rhipicephalus microplus* ticks (MH762077) and cattle (MG869510) from China and Korean water deer (LC432114; Table S3). Although only two goat sequences were generated through amplification of the *A. capra msp4* gene (Figure 5), the results of the phylogenetic classification were identical to those shown by amplification of the *16S rRNA* gene (Figure 3). In other words, all *A. capra* sequences of these two genes were classified into an independent clade clearly distinct from other members of *Anaplasma* species. The *msp4* sequences showed 100% identity with the sequences isolated from Korean water deer (LC432231), dogs (MK838607), and humans (KM206277) from China (Table S4).

**Figure 6:**
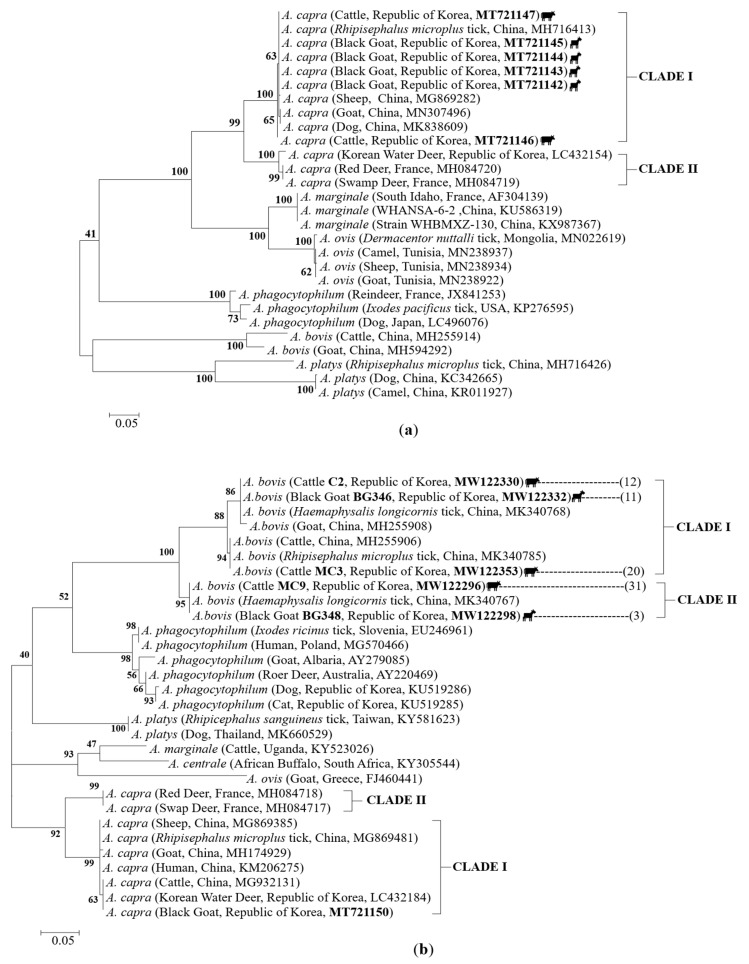
Phylogenetic tree based on the (**a**) *gltA* and (**b**) *groEL* gene fragments of the *Anaplasma* species. The phylogenetic tree was constructed using the neighbor-joining method with 1000 replicates of the alignment (MEGA 6.06 software). The numbers of nucleotides were 480 bp and 238 bp in the final alignment for *gltA* and *groEL* genes, respectively. Cattle and goat icons indicate the sequences found in this study. The numbers in brackets represent the total number of sequences that are identical to the representative sequence. Isolate, country, and GenBank accession numbers are shown in parentheses. The scale bar represents the number of nucleotide substitutions between sequences. Clades are denoted by roman numbers. Numbers on the branches indicate percent support for each clade.

In contrast, phylogenetic analysis of *A. capra* based on *gltA* and *groEL* genes classified the sequences into two major clades (Figure 6). Sequence analysis of the *A. capra gltA* gene revealed two different variants in this study. Five isolates (MT721147, MT721145, MT721144, MT721143, and MT721142) were found to be 100% identical to those obtained from Chinese *R. microplus* ticks (MH716413), while the isolate MT721146 obtained from a cattle showed an identity of 99.8% (with one substitution, G/A at position 460) with Chinese tick isolates (MH716413), and 99.6% with the rest of the clustered sequences, which were isolated from Chinese goats, dogs, and sheep (Figure 6a, Table S5), suggesting that this is a novel variant of the *A. capra gltA* gene, which has never been reported before (Figure S1).

For the *A. capra groEL* gene, one sequence was acquired from a goat (MT721150). This sequence shared 100% identity with the isolates from Chinese cattle (MG932131) and Korean water deer (LC432184) belonging to clade I, together with samples isolated from humans (99.6%, KM206275), sheep (99.6%, MG869385), *R. microplus* ticks (99.6%, MG869481), and goats (99.2%, MH174929) from China (Figure 6b, Table S6).

For *A. bovis groEL* gene sequences, three different variants were obtained: variant I (represented by the isolates C2 [cattle] and BG346 [goat]), variant II (represented by the isolate MC3 [cattle]), and variant III (represented by the isolates MC9 [cattle] and BG348 [goat]). Variant I shared 100% identity with *H. longicornis* ticks (MK340768), while variant II was 100% identical to *R. microplus* ticks (MK340785). Both variants belonged to clade I together with Chinese isolates. Finally, variant III showed 100% identity with the sequences from Chinese *H. longicornis* ticks (MK340767), thus representing the second clade (Figure 6b, Table S7).

## 3. Discussion

In recent years, environmental factors such as global warming and deforestation have favored the increase in tick populations due to changes in their seasonal activity. However, the introduction of new animal species acting as a potential reservoir of one or even multiple pathogen species has led to a rapid distribution and expansion in the number of ticks. This phenomenon can be exemplified by anaplasmosis, a tick-borne disease distributed worldwide that has evolved in its pathogenesis over time. Despite recent reports of new species belonging to the genus *Anaplasma* and the considerable increase in their zoonotic potential, few studies on *Anaplasma* infection have been carried out in the ROK. The present study was mainly focused on investigating the genetic variability and prevalence of *Anaplasma* species as single and multiple infection in cattle and goats from different provinces of the ROK.

Our investigation revealed that the Korean cattle were mainly infected with *A. phagocytophilum* (21.1%, 81/384) instead of *A. bovis*, whose main hosts are supposed to be cows and buffalo, since its first identification in cattle in 1936 [23]. Of the 81 cattle infected with *A. phagocytophilum*, 80 were from Gyeongsangbuk-do province. Conversely, a study carried out in 2016 in the same province identified *A. phagocytophilum* with a relatively low infection rate (4.7%) [18] compared with the results obtained in our study (88.9%). *A. bovis* was the second dominant pathogen (18.2%, 70/384) identified in this study. Based on the geographic distribution, Gyeongsangbuk-do was also the province with a higher number of carriers of *A. bovis* (74.4%). Our results differed from those of previous molecular studies conducted in the ROK, which reported a comparatively low prevalence of *A. bovis* in cattle: 4.2% (3/71) in Jeju Island [12], 1.9% (11/581) in Gyeongsangnam-do, and 0.2% (1/638) in Gyeongsangbuk-do province [13]. This sudden increase in *Anaplasma* spp. prevalence rates may be largely associated with climate change. It is known that the Korean peninsula is gradually transitioning to a subtropical climate [13], where a hot summer season and changes in rainfall patterns can favor the abundance and spread of ticks to different localities.

In addition, the present study also detected *A. capra* infection in cattle. In 2018, *A. capra* was described for the first time in Korean cattle from Gyeongsangnam-do with a low infection rate (0.4%) [13]. These results are similar to those obtained in this study (0.5%). An important point to consider is that one cow positive for *A. capra* was infected with *A. phagocytophilum* and *A. bovis*, which is the first detection of triple infection with *Anaplasma* species in the ROK. It should also be noted that this is the first molecular study of co-infection cases caused by several *Anaplasma* species in cattle, in which triple and doble infection cases were detected, with the latter type involving *A. phagocytophilum* and *A. bovis*. Our findings, along with previous studies performed in the ROK [13,18], demonstrates that the cattle residing in provinces located at a lower latitude, such as Gyeongsangbuk-do and Gyeongsangnam-do, are naturally infected with *Anaplasma* spp., thus suggesting that cattle may play an important role in the enzootic maintenance of *Anaplasma* infection.

On the other hand, goats were found to be carriers of two *Anaplasma* species: *A. bovis* (7.3%) and *A. capra* (1.3%). The results obtained in the current study coincide with those previously described in Ulsan Metropolitan City, ROK, which reported infection rates of 8.6% and 2.2% for *A. bovis* and *A. capra*, respectively [16]. Interestingly, we also found three co-infection cases due to *A. bovis* and *A. capra*, two of which were in Jeollabuk-do and the other in Jeollanam-do province. Thus, our findings shed light on co-infection types that may be found among Korean goats. Although *A. phagocytophilum* and *A. ovis* were not identified in our study, we cannot rule them out as causative agents of caprine anaplasmosis, since a study conducted in China demonstrated the prevalence of these species, even reporting cases of triple infection with *A. ovis*, *A. bovis*, and *A. phagocytophilum* [15]. It is also worth mentioning that a serological study conducted in the ROK detected antibodies against the major surface protein 5 (*msp5*) of *A. marginale*, *A. centrale*, and *A. ovis*, using a commercial competitive enzyme-linked immunosorbent assay (ELISA). The seroprevalence rate reported in this study was 6.6% (36/544) in native Korean goats [30]. However, identification of a species among *A. marginale*, *A. centrale*, and *A. ovis* was not performed by PCR; these findings compared with our data suggest that *Anaplasma* infection in goats could be caused by other *Anaplasma* species, in addition to *A. bovis* and *A. capra* pathogens. Additional studies are necessary to corroborate this finding, taking into consideration a large sample size that involves the different Korean provinces.

Despite the fact that the genes that are most often targeted to investigate the genetic diversity of *A. phagocytophilum* involve the *16S rRNA* locus, *groESL* operon, major surface protein coding genes (*msp2* and *msp4*), and *ank*A gene [23], PCR amplification of the *msp2* and *ank*A genes was unsuccessful in the present study. This could be due to the complex epidemiological cycles of *A. phagocytophilum*, which involves different genetic variants, vectors, and host species [23]. However, according to the results obtained by the amplification of the *A. phagocytophilum 16S rRNA* and its phylogenetic analysis, we demonstrated that five novel strains are circulating among Korean cattle. In addition, the multilocus genotyping of *A. capra* not only favored the ratification of cattle as a potential reservoir of this *Anaplasma* species but also contributed to the identification of a new variant of the *A. capra gltA* gene, thus aiding in elucidation of the genetic variability of *A. phagocytophilum* and *A. capra*, two species with zoonotic importance.

## 4. Material and Methods

### 4.1. Sample Collection

A total of 686 whole blood samples from domestic ruminants (384 cattle and 302 black goats) were collected from different provinces of the ROK, which were randomly selected between August 2015 and June 2020. These samples were collected in sterile 10 mL tubes containing EDTA anticoagulant and transported to the laboratory in an icepack container. Goat blood samples were collected in Jeollabuk-do (*n* = 37), Gwangju Metropolitan City (*n* = 41), and Jeollanam-do (*n* = 224) province, while cattle blood samples were collected in Gyeongsangbuk-do (*n* = 90), Gyeongsangnam-do (*n* = 65), Jeollanam-do (*n* = 65), Gyeonggi-do (*n* = 50), Chungbuk-do (*n* = 54), and Chungnam-do (*n* = 60) provinces (Figure 1).

### 4.2. DNA Extraction

Genomic DNA was extracted from 200 µL whole blood samples using a commercial LaboPass DNA Purification Kit (Cosmo Genetech, Seoul, Korea) according to the manufacturer’s instructions. The extracted DNA was stored at −20 °C until further analysis. The quantity and purity of the extracted DNA were calculated using a NanoDrop spectrophotometer (Implen NanoPhotometer Classic, Germany).

### 4.3. PCR Amplification

DNA samples were subjected to nested PCR (nPCR) to amplify the *16S rRNA* gene fragments of *A. phagocytophilum* and *A. bovis*. The first round was performed using the primer pair AE1-F/AE1-R, which amplifies the *16S rRNA* gene shared by all *Anaplasma* spp. (Table 3). In the second round, PCR-positive samples were reamplified by employing species-specific primer sets EE3/EE4 and ABKf/AB1r for *A. phagocytophilum* [31] and *A. bovis* [32], respectively. For multilocus genotyping, *A. phagocytophilum msp2* [33] and *ank*A [34] gene fragments were amplified using nPCR, and *A. bovis groEL* gene using semi-nested PCR [35]. For *A. capra*, the *16S rRNA* [7], *gltA* [36], *groEL*, and *msp4* [37] genes were amplified using the primer sets described in Table 3. The *A. ovis msp4* gene was amplified using single-step PCR with the primers pairs MSP45/MSP43 [38]. These reactions were performed in a total volume of 30 µL, containing 10 pmol of each primer, 15 µL of 2x Taq PCR Pre-mix (BioFACT, Daejeon, Korea), 50 to 100 ng of genomic DNA samples for the first round of PCR, and 1 µL of the first PCR product was used as template DNA for the nPCR. Each reaction was conducted in a SimpliAmp Thermal Cycler (Thermo Fisher Scientific, Korea) under optimal cycling conditions (Table 3). The PCR products were visualized under UV light after 1.5% agarose gel electrophoresis, using a 100 bp ladder (SiZer-100 DNA Marker Solution, iNtRON Biotechnology, Korea) as a DNA size marker.

### 4.4. DNA Sequencing and Phylogenetic Analysis

The PCR products were purified using the DNA Gel Extraction S & V Kit (BIONICS, Daejeon, ROK) and were directly sequenced using an Applied Biosystems 3730 DNA Analyzer (Thermo Fisher Scientific, Foster City, USA). The obtained sequences were evaluated with Chromas software, aligned using the multiple sequence alignment program ClustalX 2.1, compared with reference sequences searched in the NCBI, analyzed using the Basic Local Alignment Search Tool to determine the identity percentage between them, and finally examined with a similarity matrix. Relationships between individuals were assessed by the neighbor-joining method with nucleotide distance (*p* distance) for 1000 replications with a bootstrap analysis. A phylogenetic tree was constructed based on nucleotide alignment using MEGA 6.06 software.

### 4.5. Nucleotide Sequence Accession Numbers

The sequences obtained in this study have been deposited in the GenBank database with the following accession numbers: *A. phagocytophilum 16S rRNA* (MT754291 to MT754365), *A. bovis 16S rRNA* (MT754858 to MT754934), *A. bovis groEL* (MW122296 to MW122372), *A. capra 16S rRNA* (MT798599 to MT798604), *A. capra msp4* (MT721148 to MT721149), *A. capra gltA* (MT721142 to MT721147), and *A. capra groEL* (MT721150).

## 5. Conclusions

Based on the results obtained in this study, in the ROK, cattle are mainly infected with *A. phagocytophilum*, while goats act mainly as carriers of *A. bovis*. This study has contributed to the ratification of cattle as a potential reservoir of the emerging zoonotic pathogen, *A. capra*. Although *A. ovis* was not detected, we cannot rule it out as a causative agent of bovine and caprine anaplasmosis; further studies are needed to corroborate this finding. Our study sheds light on the geographical distribution of *Anaplasma* infection types; however, supplementary studies are needed to clarify the clinical presentation and epidemiological significance of genetic variants of *Anaplasma* species to establish effective prevention and control strategies.

## Figures and Tables

**Table 1:** Prevalence of single infection and co-infection cases with *Anaplasma* species detected in cattle and goat blood samples.

Animal Species	No. Tested	No. of Positive (IR ^1^, %)	Type of Infection Identified in Tested Blood Samples							Not Infected (%)
			Single Infection				Double Infection		Triple Infection	
			*A.* *phago*^2^ (%)	*A.* *bovis* (%)	*A.* *capra* (%)	*A.* *ovis* (%)	*A. bovis* + *A. phago* ^2^ (%)	*A. bovis* + *A. capra* (%)	*A. phago*^2^ + *A. bovis* + *A. capra* (%)	
Cattle	384	87 (22.7)	16 (4.2)	5 (1.3)	1 (0.3)	0 (0)	64 (16.7)	0 (0)	1 (0.3)	297 (77.3)
Goat	302	23 (8.0)	0 (0)	19 (6.3)	1 (0.3)	0 (0)	0 (0)	3 (1.0)	0 (0)	279 (94.4)
Total	686	110 (16.0)	16 (2.3)	24 (3.5)	2 (0.3)	0 (0)	64 (9.3)	3 (0.4)	1 (0.1)	576 (83.9)

^1^ IR, infection rate; ^2^ *A. phago*: *Anaplasma phagocytophilum*.

**Table 2:** Prevalence rate per *Anaplasma* species analyzed in domestic ruminants in the Republic of Korea.

Host	Collected Province	No. Tested	*A. phago*^1^ (%)	*A. bovis* (%)	*A. capra* (%)	*A. ovis* (%)
Cattle	Gyeongsangbuk-do	90	80 (88.9)	67 (74.4)	1 (1.1)	0 (0)
	Gyeongsangnam-do	65	0 (0)	0 (0)	0 (0)	0 (0)
	Jeollanam-do	65	0 (0)	1 (1.5)	0 (0)	0 (0)
	Gyonggi-do	50	0 (0)	0 (0)	0 (0)	0 (0)
	Chungbuk-do	54	1 (1.9)	1 (1.9)	1 (1.9)	0 (0)
	Chungnam-do	60	0 (0)	1 (1.7)	0 (0)	0 (0)
	***Subtotal***	384	81 (21.1)	70 (18.2)	2 (0.5)	0 (0)
Goat	Jeollabuk-do	37	0 (0)	10 (27.0)	2 (5.4)	0 (0)
	Gwangju Metropolitan City	41	0 (0)	3 (7.3)	0 (0)	0 (0)
	Jeollanam-do	224	0 (0)	9 (4.0)	2 (1.0)	0 (0)
	***Subtotal***	302	0 (0)	22 (7.3)	4 (1.3)	0 (0)
**Grand total**		686	81 (11.8)	92 (13.4)	6 (0.9)	0 (0)

^1^*A. phago*: *Anaplasma phagocytophilum*.

**Table 3:** Oligonucleotide primers and polymerase chain reaction (PCR) conditions used in this study.

Species	Target Gene	Primer Name and PCR Conditions	Primer Sequences (5′-3′)			Cycles	Amplicon Size (bp)	References
			Denaturation (°C/min)	Annealing (°C/min)	Extension (°C/min)			
*Anaplasma* spp.	*16S rRNA* ^1^	*AE1-F*	AAGCTTAACACATGCAAGTCGAA			35	1406	Oh et al. (2009) [27]
		*AE1-R*	AGTCACTGACCCAACCTTAAATG					
		Conditions	94/1	56/1	72/1.5			
*A. phagocytophilum*	*16S rRNA* ^2^	*EE3*	GTCGAACGGATTATTCTTTATAGCTTGC			25	926	Barlough et al. (1996) [31]
		*EE4*	CCCTTCCGTTAAGAAGGATCTAATCTCC					
		Conditions	94/0.50	56/0.50	72/0.75			
	*msp2*	*msp2fullF*	TCAGAAAGATACACGTGCGCCC			35	1079	Lin et al. (2004) [33]
		*msp2fullR*	TTATGATTAGGCCTTTGGGCATG					
		Conditions	94/1	54/1	72/1			
		*msp2F*	GGTTACATAAGGGCCGCAAAGGTG			25	467	
		*msp2R*	CCGGCGCATGTGTAAGGTGAAA					
		Conditions	94/0.5	57/0.5	72/0.5			
	*ank*A	*U7*	GCGTCTGTAAGGCAGATTGTG			35	1696	Massung et al. (2000) [34]
		*1R1*	TATACACCTGGAGTAGGAAC					
		Conditions	94/1	57/1	72/1.5			
		*U8*	TAAGATAGGTTTAGTAAGACG			25	460	
		*1R7*	TGCATCGTCATTACGCACAAGGTC					
		Conditions	94/0.75	57/0.75	72/0.75			
*A. bovis*	*16S rRNA* ^3^	*ABKf*	TAGCTTGCTATGGGGACAA			25	547	Kang et al. (2011) [32]
		*AB1r*	TCTCCCGGACTCCAGTCTG					
		Conditions	94/0.5	59/0.5	72/0.5			
	*groEL*	*bovis-groEL-F1*	GTTCGCAGTATTTTGCCAGT			30	845	Guo et al. (2019) [35]
		*bovisgroEL-R*	CTGCRTTCAGAGTCATAAATAC					
		*bovis-groEL-F2*	ATCTGGAAGRCCACTATTGAT					
		Conditions	94/0.7	56/0.7	72/1			
*A. capra*	*16S rRNA*	*Forward*	TTGAGAGTTTGATCCTGGCTCAGAACG			57	1499	Li et al. (2015) [7]
		*Reverse*	WAAGGWGGTAATCCAGC					
		Conditions	94/0.75	57/0.75	72/0.75			
	*gltA*	*Outer-f*	GCGATTTTAGAGTGYGGAGATTG			30	1031	
		*Outer-r*	TACAATACCGGAGTAAAAGTCAA					
		Conditions	94/0.75	55/0.75	72/0.75			
		*Inner-f*	TCATCTCCTGTTGCACGGTGCCC			30	594	Yang et al. (2016) [36]
		*Inner-r*	CTCTGAATGAACATGCCCACCCT					
		Conditions	94/0.75	60/0.75	72/0.75			
	*groEL*	*Forward*	TGAAGAGCATCAAACCCGAAG			30	874	Yang et al. (2017) [37]
		*Reverse*	CTGCTCGTGATGCTATCGG					
		Conditions	94/0.75	55/0.75	72.0.75			
	*msp4*	*Forward*	GGGTTCTGATATGGCATCTTC			30	656	
		*Reverse*	GGGAAATGTCCTTATAGGATTCG					
		Conditions	94/0.75	53/0.75	72/0.75			
*A. ovis*	*msp4*	*MSP45*	GGGAGCTCCTATGAATTACAGAGAATTGTTTAC			35	852	De la Fuente et al. (2007) [38]
		*MSP43*	CCGGATCCTTAGCTGAACAGGAATCTTGC					
		Conditions	94/0.5	60/0.5	68/1			

^1^ Primer pair used in the first round for the amplification of the *16S rRNA* gene shared by all *Anaplasma* spp., ^2^ species-specific primer sets used in the second round for the amplification of the *16S rRNA* gene of *A. phagocytophilum*, and ^3^ species-specific primer sets used in the second round for the amplification of the *16S rRNA* gene of *A. bovis*.

## Data Availability

The authors confirm that the data supporting the findings of this study are available within the article and its supplementary materials.

## Supplementary Materials

The following are available online at https://www.mdpi.com/2076-0817/10/1/28/s1, Figure S1: DNA sequencing electropherogram, Table S1: Genetic identity matrix based on the *16S rRNA* gene fragments of *A. phagocytophilum* (547 bp), Table S2: Genetic identity matrix based on the *16S rRNA* gene fragments of *A. bovis* (547 bp), Table S3: Genetic identity matrix based on the *16S rRNA* gene fragments of *A. capra* (547 bp), Table S4: Genetic identity matrix based on the *msp4* gene fragments of *A. capra* (527 bp), Table S5: Genetic identity matrix based on the *gltA* gene fragments of *A. capra* (480 bp), Table S6: Genetic identity matrix based on the *groEL* gene fragments of *A. capra* (238 bp), Table S7: Genetic identity matrix based on the *groEL* gene fragments of *A. bovis* (238 bp).
