# Supplementary material for: Co-Infection with Anaplasma Species and Novel Genetic Variants Detected in Cattle and Goats in the Republic of Korea
Source: Pathogens. 2021 Jan 1;10(1):28. doi: 10.3390/pathogens10010028 (PMC7830860; doi:10.3390/pathogens10010028)
Supplement: Supplementary file 1 [file pathogens-10-00028-s001.pdf]

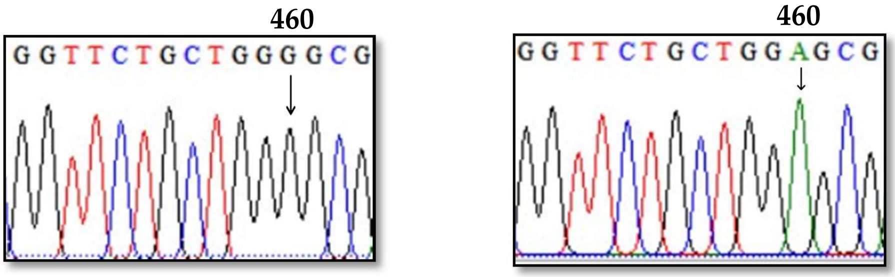

(**a**) (**b**)

Figure S1. DNA sequencing electropherogram. Arrows indicate the point mutation found in the obtained *A. capra gltA* sequences, which was variation G/A at position 460. (**a**) Indicates the sequences obtained from cattle isolates MT721147, MT721145, MT721144, MT721143, and MT721142. (**b**) Represents the novel variant found in cattle blood samples (MT721146)

Table S1. Genetic identity matrix based on the *16S rRNA* gene fragments of *A. phagocytophilum* (547 bp).

No.	Isolate	Country	GenBank accession number	No.	1	2	3	4	5	6	7	8	9	10	11	12	13	14
**1**	Cattle [1]	ROK [2]	MT754296, MT754301	**1**	**100**	99.6	99.6	99.6	99.5	99.5	98.5	98.5	98.5	98.5	98.4	98.2	98.0	98.0
**2**	Cattle [1]	ROK [2]	MT754292	**2**	2	**100**	99.6	99.6	99.8	99.5	98.4	98.4	98.4	98.4	98.0	98.2	98.0	98.0
**3**	*H. longicornis* tick	North Korea	KC422267	**3**	2	2	**100**	100	99.5	99.5	98.5	98.5	98.5	98.5	98.4	98.5	98.4	98.4
**4**	Wild Deer	Japan	AB196721	**4**	2	2	0	**100**	99.5	99.5	98.5	98.5	98.5	98.5	98.4	98.5	98.4	98.4
**5**	Cattle [1]	ROK [2]	MT754291	**5**	3	1	3	3	**100**	99.6	98.2	98.2	98.2	98.2	97.8	98.0	98.2	98.2
**6**	Cattle [1]	ROK [2]	MT754302	**6**	3	1	3	3	2	**100**	98.2	98.2	98.2	98.2	97.8	98.0	97.8	97.8
**7**	Rat	China	DQ458807	**7**	8	9	8	8	10	10	**100**	100	100	100	99.6	99.5	99.3	99.3
**8**	Cat	ROK [2]	KR021166	**8**	8	9	8	8	10	10	0	**100**	100	100	99.6	99.5	99.3	99.3
**9**	Dog	ROK [2]	KU513794	**9**	8	9	8	8	10	10	0	0	**100**	100	99.6	99.5	99.3	99.3
**10**	Human	ROK [2]	MF582329	**10**	8	9	8	8	10	10	0	0	0	**100**	99.6	99.5	99.3	99.3
**11**	Goat	China	HQ872465	**11**	9	11	9	9	12	12	2	2	2	2	**100**	99.8	99.6	99.6
**12**	Cattle	Turkey	KP745629	**12**	10	10	8	8	11	11	3	3	3	3	1	**100**	99.8	99.8
**13**	Horse	Sweden	AY527214	**13**	11	11	9	9	10	12	4	4	4	4	2	1	**100**	100
**14**	Korean Water Deer	ROK [2]	GU556624	**14**	11	11	9	9	10	12	4	4	4	4	2	1	0	**100**

Isolate, country, and GenBank accession numbers of each sequence are specified in the left site of the matrix. White boxes of the matrix indicate the percent identity between sequences and gray boxes shown the nucleotide differences between them. [1] Representative sequences of *A. phagocytophilum 16S rRNA* gene obtained in this study. [2] ROK, Republic of Korea.

Table S2. Genetic identity matrix based on the *16S rRNA* gene fragments of *A. bovis* (547 bp).

No.	Isolate	Country	GenBank accession number	No.	1	2	3	4	5	6	7	8
**1**	Black Goat [1]	ROK [2]	MT754932	**1**	**100**	99.8	99.8	99.8	99.5	99.1	98.9	98.0
**2**	Cattle [1]	ROK [2]	MT754879	**2**	1	**100**	100	100	99.3	99.3	99.1	98.2
**3**	Black Goat [1]	ROK [2]	MT754934	**3**	1	0	**100**	100	99.3	99.3	99.1	98.2
**4**	Goat	China	MH255939	**4**	1	0	0	**100**	99.3	99.3	99.1	98.2
**5**	Cattle	China	MH255941	**5**	3	4	4	4	**100**	99.3	99.1	98.2
**6**	Dog	Japan	HM131218	**6**	5	4	4	4	4	**100**	99.5	98.2
**7**	Monkey	Malaysia	KM114613	**7**	6	5	5	5	5	3	**100**	98.4
**8**	*A. triguttatum* tick	Australia	KY425447	**8**	11	10	10	10	10	10	9	**100**

Isolate, country, and GenBank accession numbers of each sequence are specified in the left site of the matrix. White boxes of the matrix indicate the percent identity between sequences and gray boxes shown the nucleotide differences between them. [1] Representative sequences of *A. bovis 16S rRNA* gene obtained in this study. [2] ROK, Republic of Korea.

Table S3. Genetic identity matrix based on the *16S rRNA* gene fragments of *A. capra* (547 bp).

No.	Isolate	Country	GenBank accession number	No.	1	2	3	4	5	6
**1**	Black Goat [1]	ROK [2]	MT798599	**1**	**100**	100	100	100	100	99.8
**2**	Cattle [1]	ROK [2]	MT798603	**2**	0	**100**	100	100	100	99.8
**3**	*R. microplus* tick	China	MH762077	**3**	0	0	**100**	100	100	99.8
**4**	Cattle	China	MG869510	**4**	0	0	0	**100**	100	99.8
**5**	Korean Water Deer	ROK [2]	LC432114	**5**	0	0	0	0	**100**	99.8
**6**	Sheep	China	MG869508	**6**	1	1	1	1	1	**100**

Isolate, country, and GenBank accession numbers of each sequence are specified in the left site of the matrix. White boxes of the matrix indicate the percent identity between sequences and gray boxes shown the nucleotide differences between them. [1] Representative sequences of *A. capra 16S rRNA* gene obtained in this study. [2] ROK, Republic of Korea.

Table S4. Genetic identity matrix based on the *msp4* gene fragments of *A. capra* (527 bp).

No.	Isolate	Country	GenBank accession number	No.	1	2	3	4	5	6
**1**	Black Goat [1]	ROK [2]	MT721149, MT721148	**1**	**100**	100	100	100	99.8	99.4
**2**	Korean Water Deer	ROK [2]	LC432231	**2**	0	**100**	100	100	99.8	99.4
**3**	Dog	China	MK838607	**3**	0	0	**100**	100	99.8	99.4
**4**	Human	China	KM206277	**4**	0	0	0	**100**	99.8	99.4
**5**	*H. longicornis* tick	China	KR261640	**5**	1	1	1	1	**100**	99.2
**6**	Goat	China	MH174933	**6**	3	3	3	3	4	**100**

Isolate, country, and GenBank accession numbers of each sequence are specified in the left site of the matrix. White boxes of the matrix indicate the percent identity between sequences and gray boxes shown the nucleotide differences between them. [1] Representative sequences of *A. capra msp4* gene obtained in this study. [2] ROK, Republic of Korea.

Table S5. Genetic identity matrix based on the *gltA* gene fragments of *A. capra* (480 bp).

No.	Isolate	Country	GenBank accession number	No.	1	2	3	4	5	6	7	8	9	10
**1**	Black Goat [1]	ROK [2]	MT721145	**1**	**100**	100	100	99.8	99.8	99.8	99.8	88.1	88.1	87.9
**2**	Cattle [1]	ROK [2]	MT721147	**2**	0	**100**	100	99.8	99.8	99.8	99.8	88.1	88.1	87.9
**3**	*R. microplus* tick	China	MH716413	**3**	0	0	**100**	99.8	99.8	99.8	99.8	88.1	88.1	87.9
**4**	Cattle [1]	ROK [2]	MT721146	**4**	1	1	1	**100**	99.6	99.6	99.6	88.3	88.3	88.1
**5**	Sheep	China	MG869282	**5**	1	1	1	2	**100**	99.6	99.6	87.9	87.9	87.7
**6**	Goat	China	MN307496	**6**	1	1	1	2	2	**100**	100	87.9	87.9	87.7
**7**	Dog	China	MK838609	**7**	1	1	1	2	2	0	**100**	87.9	87.9	87.7
**8**	Red Deer	France	MH084720	**8**	57	57	57	56	58	58	58	**100**	100	98.1
**9**	Swap Deer	France	MH084719	**9**	57	57	57	56	58	58	58	0	**100**	98.1
**10**	Korean Water Deer	ROK [2]	LC432154	**10**	58	58	58	57	59	59	59	9	9	**100**

Isolate, country, and GenBank accession numbers of each sequence are specified in the left site of the matrix. White boxes of the matrix indicate the percent identity between sequences and gray boxes shown the nucleotide differences between them. [1] Representative sequences of *A. capra gltA* gene obtained in this study. [2] ROK, Republic of Korea.

Table S6. Genetic identity matrix based on the *groEL* gene fragments of *A. capra* (238 bp).

No.	Isolate	Country	GenBank accession number	No.	1	2	3	4	5	6	7	8	9
**1**	Black Goat [1]	ROK [2]	MT721150	**1**	**100**	100	100	99.6	99.6	99.6	99.2	91.2	91.2
**2**	Cattle	China	MG932131	**2**	0	**100**	100	99.6	99.6	99.6	99.2	91.2	91.2
**3**	Korean Water Deer	ROK [2]	LC432184	**3**	0	0	**100**	99.6	99.6	99.6	99.2	91.2	91.2
**4**	Human	China	KM206275	**4**	1	1	1	**100**	100	100	99.6	91.2	91.2
**5**	Sheep	China	MG869385	**5**	1	1	1	0	**100**	100	99.6	91.2	91.2
**6**	*R. microplus* tick	China	MG869481	**6**	1	1	1	0	0	**100**	99.6	91.2	91.2
**7**	Goat	China	MH174929	**7**	2	2	2	1	1	1	**100**	90.8	90.8
**8**	Red Deer	France	MH084718	**8**	21	21	21	21	21	21	22	**100**	100
**9**	Swap Deer	France	MH084717	**9**	21	21	21	21	21	21	22	0	**100**

Isolate, country, and GenBank accession numbers of each sequence are specified in the left site of the matrix. White boxes of the matrix indicate the percent identity between sequences and gray boxes shown the nucleotide differences between them. [1] Representative sequences of *A. capra groEL* gene obtained in this study. [2] ROK, Republic of Korea.

Table S7. Genetic identity matrix based on the *groEL* gene fragments of *A. bovis* (238 bp).

No.	Isolate	Country	GenBank accession number	No.	1	2	3	4	5	6	7	8	9
**1**	Cattle [1]	ROK [2]	MW122330	**1**	100	100	100	99.2	98.0	98.0	98.0	91.2	91.2
**2**	Black Goat [1]	ROK [2]	MW122332	**2**	0	100	100	99.2	99.8	98.0	98.0	91.2	91.2
**3**	*H. longicornis* tick	China	MK340768	**3**	0	0	100	99.2	99.8	98.0	98.0	91.2	91.2
**4**	Goat	China	MH255908	**4**	2	2	2	100	97.1	97.1	97.1	90.3	90.3
**5**	Cattle	China	MH255906	**5**	5	5	5	7	100	100	100	92.4	92.4
**6**	*R. microplus* tick	China	MK340785	**6**	5	5	5	7	0	100	100	92.4	92.4
**7**	Cattle [1]	ROK [2]	MW122353	**7**	5	5	5	7	0	0	100	92.4	92.4
**8**	Cattle [1]	ROK [2]	MW122296	**8**	21	21	21	23	18	18	18	100	100
**9**	Tick	China	MK340767	**9**	21	21	21	23	18	18	18	0	100
**10**	Black Goat [1]	ROK [2]	MW122298	**10**	21	21	21	23	18	18	18	0	0

Isolate, country, and GenBank accession numbers of each sequence are specified in the left site of the matrix. White boxes of the matrix indicate the percent identity between sequences and gray boxes shown the nucleotide differences between them. [1] Representative sequences of *A. bovis groEL* gene obtained in this study. [2] ROK, Republic of Korea.
